# Retina and glaucoma: surgical complications

**DOI:** 10.1186/s40942-018-0135-x

**Published:** 2018-09-05

**Authors:** Niroj Kumar Sahoo, Pasyanthi Balijepalli, Sumit Randhir Singh, Mahima Jhingan, Sirisha Senthil, Jay Chhablani

**Affiliations:** 1 Smt. Kanuri Santhamma Centre for Vitreo-Retinal Diseases, L V Prasad Eye Institute, Hyderabad, 500034 India; 2 VST Center for Glaucoma Services, L V Prasad Eye Institute, Hyderabad, 500034 India; 3 Aravind Eye System, Madurai, India

**Keywords:** Retinal complications, Glaucoma, Filtration surgery, Glaucoma after retinal surgeries

## Abstract

**Background:**

The close structural and microcirculatory co-relation between anterior and posterior segments of eye make them very vulnerable to complications when one of them is affected surgically. With the advent of anti-fibrotic agents in the management of glaucoma, the rates of vitreoretinal complications have become more frequent.

**Main body:**

Common retinal complications after glaucoma surgeries include choroidal detachment; ocular decompression retinopathy; haemorrhagic choroidal detachment; hypotony maculopathy; malignant glaucoma; vitreous haemorrhage; bleb endophthalmitis; retinal detachment. Similarly, intraocular pressure rise is often noted after scleral buckle; pars plana vitrectomy; intravitreal gas injection; silicone oil injection; intravitreal steroid injection.

**Conclusion:**

The article provides some insight into some of the complications after glaucoma and retina surgeries, including the pathogenetic mechanisms behind each complication and available management options.

## Background

It is very common to see retina and glaucoma surgeon, working in tandem to manage some of the most complicated cases. Vitreous, retina, choroid and aqueous humour dynamics share a complex architectural milieu. Be it due to the close proximity of the structures or the interdependent blood circulation, even the slightest of structural or microcirculatory alteration in one can lead to dramatic consequence in the other. This intrinsic co-relation often manifests itself as various complications when surgically disturbed. Whether it is an acute rise in intraocular pressure (IOP) following a retinal surgery or some of the sight threatening complications of filtration surgeries, outcomes can be relentless and unforgiving for both patient and treating physician.

This review article describes pathogenic mechanisms, clinical features, and management strategies for retinal complications after glaucoma surgeries and vice versa.

## Retinal complications following glaucoma surgery

Despite modern advances in filtration surgeries, retinal complications remain a significant cause of post operative vision loss. A sudden drop in pressure and the resultant change in ocular dynamics are implicated in most cases. Among newer minimally invasive glaucoma surgeries (MIGS), schlemm canal based micro-stent surgeries have lower rates of complications owing to the limitation in intra-ocular pressure drop due to resistance in the episcleral vessels [1]. Complications can be classified as shown in Table 1.

**Table 1:** Summary of complications following retinal and glaucoma surgeries

Conditions	Articles	Type of article	Summary
*Retinal complications of glaucoma surgeries*			
Ocular decompression retinopathy (ODR)	Fechner et al. [2]	Original article	Coined the term ocular decompression retinopathy
	Arévalo et al. [9]	Original article	ODR occurred after trabeculectomy (4 eyes), phacomulsification (3 eyes), Ahmed valve placement (1 eye), silicone oil removal (1 eye) and vitrectomy (1 eye)
	Li et al. [8]	Case report	ODR following canaloplasty
Serous choroidal detachment (CD)	Investigators A. (AGIS) [12]; Gedde et al. [13]; Elhofi and Lolah [14]	Original article Original article Original article	Incidence of 7.9–50% after penetrating glaucoma surgeries
	De Gregorio et al.[15]; Grover et al. [16]; Schlenker et al. [17]	Original article Original article Original article	CD following Xen Gel Stent
	Bakir and Pasquale [20]	Review article	Anti-metabolites as a risk factor
Suprachoroidal haemorrhage (SCH)	Tuli et al. [25]; Vaziri et al. [26]; Jeganathan et al. [27]	Original article Original article Original article	Incidence is around 0.6–1.5% after trabeculectomy and around 0.5–8.3% after tube shunt procedures
Hypotony maculopathy	Fannin et al. [31]; Tsai et al. [32]; Suner et al. [33]	Original article Original article Original article	Incidence is 1.3–20% after glaucoma filtering surgery
	Schlenker et al. [17]; Vold et al. [34]	Original article Original article	Occurs following MIGS
Malignant glaucoma	Simmons [39]; Chandler et al. [40]; Eltz and Gloor [41]	Review article Review article Review article	Incidence of 0.4–6% of cases after penetrating glaucoma surgeries
	Schlenker et al. [17]	Original article	Following Xen gel stent
Vitreous haemorrhage	Law et al. [52]	Original article	Seen in 5% of patients after aqueous shunt implantation
	Schlenker et al. [17]	Original article	Following Xen gel stent
Endophthalmitis	Ang et al. [55]; Vaziri et al. [26]; Muckley and Lehrer [57]	Original article Original article Original article	Incidence of 0.12–1.3%,
	Busbee et al. [60]	Original article	Prompt vitrectomy is considered more beneficial than tap and injections even when presenting vision is better than hand movement
Retinal detachment	Waterhouse et al. [61]	Original article	Incidence of 5% following Molteno tube implantation
*Glaucoma after retinal surgery*			
Scleral buckle	Perez et al. [65]; Smith et al. [75]; Kreiger et al. [76]; Sebestyen et al. [77]	Original article Review article Original article Original article	Incidence of angle closure glaucoma is 1.4–4.4%
	Pavlin et al. [78]	Original article	Supraciliary fluid with increased ciliary body thickness in 80% eyes following buckle
	Hayreh (1973)	Original article	Glaucoma following anterior segment ischemia
	Sidoti et al. [84]	Original article	85% success rate with the placement of silicone tube in between the buckle and the overlying capsule after incising the capsule over the buckle at the apex
	Smith et al. [85]	Original article	Krupin Denver implant and trimmed Baerveldt implants (success rates of 82 and 73% at 1 and 2 years respectively)
	Scott et al. [86]	Original article	Baerveldt implants with intact plate successfully reduces IOP
Pars plana vitrectomy	Chang [66]; Koreen et al. [87]	Original article Original article	Incidence between 11.6 and 20%
	Koreen et al. [87]	Original article	Lens extraction as a major risk factor
	Inoue et al. [91]	Original article	Success rate of trabeculectomy with Mitomycin-C is 55.1% at 1 year, and 43.1% at 3 years
Intravitreal gas injection	Vitrectomy with silicone oil or sulfur hexafluoride gas in eyes with severe proliferative vitreoretinopathy: results of a randomized clinical trial. Silicone Study Report 1 and 2	Original article	Incidence of high IOP following injection of 20% SF6 and 14% C3F8 was found to be 6.1 and 18% respectively
Silicone oil injection	Honavar et al. [68]; Unosson et al. [98]; de Corral et al. [99]	Original article Original article Original article	Incidence of glaucoma after silicone oil injection ranges from 4.8 to 48%
	Honavar et al. [68]	Original article	Pre existing glaucoma, diabetes mellitus, aphakia, rubeosis iridis, silicone oil in the anterior chamber and high IOP on first post operative day are positive risk factors whereas anatomic failure and myopia as negative risk factors for the development of glaucoma Successful IOP control in 3 out of 5 eyes that underwent trabeculectomy with mitomycin C
	Nguyen et al. [106]	Original article	60% success rate with Molteno implant
Intravitreal steroid injection	Kiddee et al. [108]; Ansari et al. [109]	Systematic review Original article	Incidence has been reported to be as high as 11–79%.
	Kiddee et al. [108]	Systematic review	IOP rise in 32% cases after 4 mg intravitreal triamcinolone, 66 and 79% following 0.59 and 2.1 mg fluocinolone implant, respectively, and 11 and 15% after 0.35 and 0.7 mg dexamethasone implant, respectively
	Ricci et al. [114]	Original article	ALT seems to be effective in reducing IOP
	Rubin et al. [115]	Original article	SLT lowered (P < 0.007) IOP in 5 eyes of 7 patients with steroid-induced increased IOP from 3 weeks to 6 months postoperative

### Ocular decompression retinopathy (ODR)

Described by Fechner et al., in 1992, it is characterized by optic nerve changes and retinal haemorrhages secondary to an iatrogenic sudden decrease in IOP after glaucoma filtering surgery [2].

#### Mechanism

Two mechanisms have been proposed for the pathogenesis. According to the mechanical theory, manifestations occur due to certain structural changes in the wall of the sclera and the resultant shearing of capillaries [3]. A sudden drop in pressure following procedure like iridotomy in an eye with pupillary block can result in rapid reduction in the posterior chamber volume and cause forward vitreous displacement and hemorrhage [4]. Also an acute drop in IOP can cause an anterior shift of lamina cribrosa leading to optic disc edema [5]. Disc edema itself can compress the central retinal vein, resulting in diffuse retinal haemorrhages.

As per the vascular theory, a transient decrease in IOP may reduce retinal arterial resistance, resulting in increased flow and leakage through already fragile capillaries [3]. A breakdown of auto-regulation mechanism occurs in conditions like poorly controlled diabetes mellitus, hypertension, prolonged exposure to elevated IOP or long-standing inflammatory conditions like uveitic glaucoma [2, 6, 7]. In these patients, a transient decrease in IOP may result in leakage through already fragile capillaries. In response to this, the vessels form fibrin plugs which appear as a white centre in the blot haemorrhages.

#### Etiology

The most common procedure implicated in ODR is trabeculectomy with Mitomycin C [5]. Procedures like iridotomy, iridoplasty, glaucoma drainage devices, trabeculotomy, anterior chamber (AC) paracentesis, vitrectomy, phacoemulsification, medical management of glaucoma, orbital decompression, deep sclerotomy, ExPRESS shunt implantation and canaloplasty have also been described [8, 9].

#### Clinical features

Although most of the cases are asymptomatic, a central scotoma or floaters may be occasionally seen. Retinal findings include white centered haemorrhages, haemorrhages involving all the retinal layers, vascular tortuosity, macular edema; disc edema, hyperemia, haemorrhage; vitreous haemorrhage and choroidal detachment. Haemorrhages can involve the peripheral retina as well as the posterior pole, and may even present with an epiretinal membrane [10].

#### Imaging

Fundus fluorescein angiography (FFA) shows blocked fluorescence in areas of haemorrhage with a normal arm-retina time. Optical coherence tomography (OCT) can be helpful in detecting macular edema [11].

#### Differential diagnosis

The differential diagnoses of ODR include central retinal vein occlusion (CRVO), ocular ischemic syndrome, Terson syndrome, diabetic retinopathy, Valsalva Retinopathy, shaken baby syndrome and coagulopathies. Older age group, venous dilatation, delayed venous filling and persistent macular edema seen in CRVO, helps in differentiating it from ODR [5].

#### Management

ODR is a self-limiting condition which usually resolves in around 2 weeks to 72 weeks. In most patients, no intervention is required. Around 14% of the patients require vitrectomy to remove a visually significant, non-resolving vitreous or pre-retinal haemorrhage. In high risk patients, a gradual control of IOP before surgery and intra-operatively has been suggested. However, if a rapid reduction is required to preserve vision, it is to be pursued, as ODR has a benign course [5].

### Serous choroidal detachment

Serous choroidal detachment (CD) is a common condition following glaucoma surgery characterized by the accumulation of serous fluid in the suprachoroidal space. The reported incidence is between 7.9 and 50% after penetrating glaucoma surgeries [12–14]. Non-penetrating glaucoma surgeries have a lower incidence due to a lower risk of post-operative hypotony. Newer MIGS procedures like sub-conjuntival Xen Gel Stent (Allergan Plc, Dublin, Ireland) have also been reported with CD [15–17].

#### Mechanism

The choriocapillaries have large fenestrations that allow proteins to escape, allowing fluid accumulation in the suprachoroidal space. Normally, the force exerted by the IOP prevents this fluid accumulation. Also, the sclera exerts an outward force, while the choroid exerts a weak inward force, resulting in a negative (− 2 mmHg) pressure gradient which allows for the passage of aqueous to the supra-choroidal space and ultimately out through the choroid or the sclera to the lymphatic orbital vessels [18]. If the IOP drops below the episcleral venous pressure, accompanied by a large inflammatory response, excessive protein leakage can occur from the choriocapillaris and a choroidal effusion can develop [19].

#### Etiology

The most common risk factor is glaucoma surgery in the setting of overfiltration or a bleb leak, especially with peri-operative antimetabolites use [20]. The added effects of cyclitis and ocular penetration of Mitomycin-C results in a decreased aqueous formation that can aggravate the process of hypotony [21]. Drugs like acetazolamide [22], can also cause a secondary acute angle closure glaucoma that may arise due to choroidal effusion. A recent comparison article between Ahmed and Baerveldt glaucoma tube shunts showed early postoperative choroidal effusion rates of 15% in Ahmed valves versus 10% in Baerveldt valves [23]. The Tube Versus Trabeculectomy (TVT) study showed a 14% prevalence of choroidal effusion in the tube group versus 13% in the trabeculectomy group [13].

#### Clinical features

**Fig. 1:**
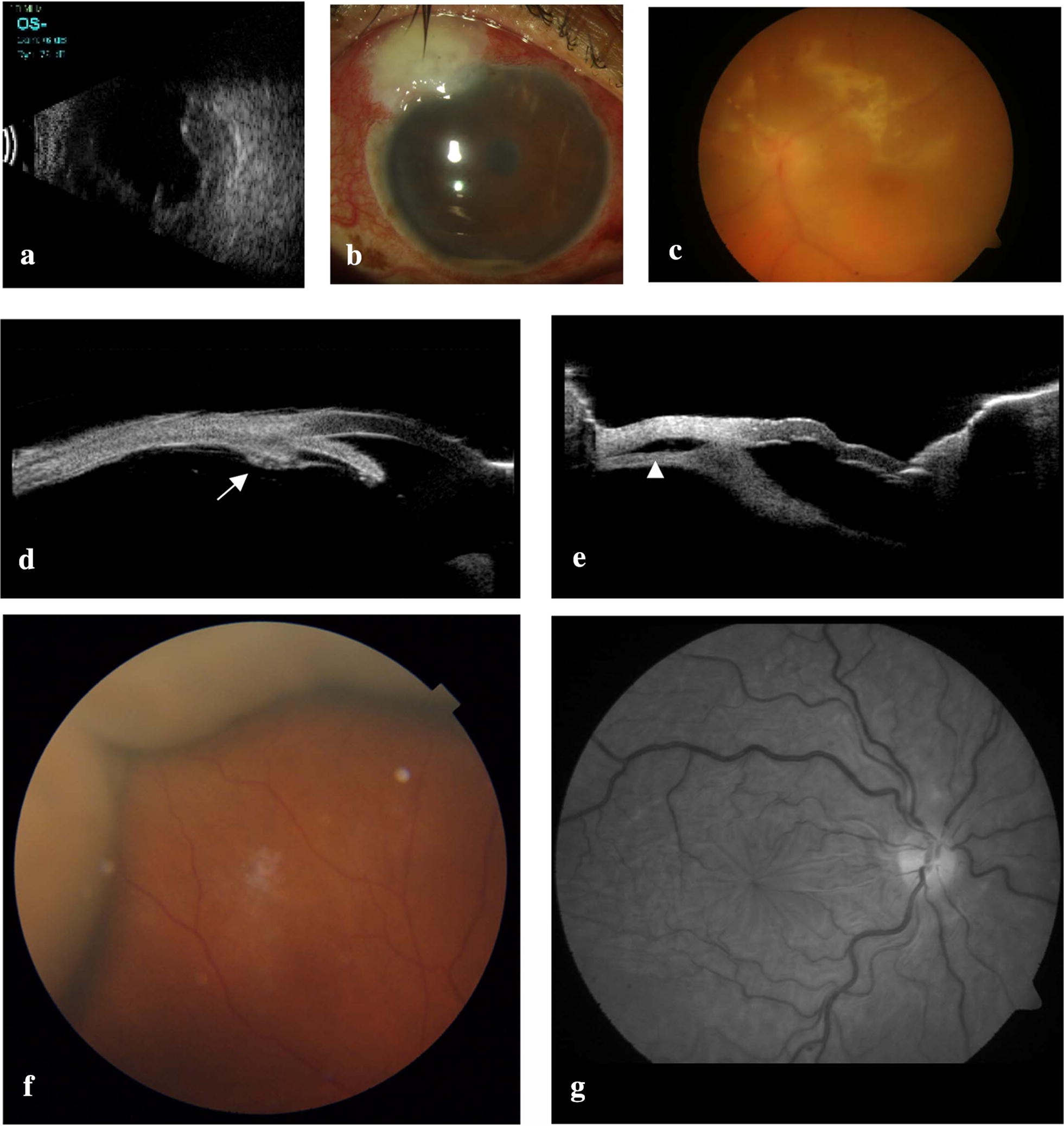
Retinal complications after glaucoma surgery: ultrasound B-scan showing supra-choroidal haemorrhage (**a**); blebitis with hypopyon (**b**); fundus image in endophthalmitis (**c**); ultrasound biomicroscopic (UBM) image showing anterior rotation of ciliary body in malignant glaucoma (arrow) (**d**), cilio-choroidal detachment (arrow-head) (**e**); fundus image showing choroidal detachment (**f**); red free image showing retinal folds in hypotony maculopathy (**g**)

It is usually self-limiting, localized or non-appositional and presents as painless, smooth, dome-shaped, orange or light brown elevation, found mostly in the peripheral fundus (Fig. 1f). Its extent is usually limited by the anatomy of the vortex veins. It demonstrates a characteristic trans-illumination. The above mentioned features help to differentiate it from retinal detachments (RD). Vision may be affected by a large CD affecting macula or due to concurrent conditions like serous RD, vitreous haemorrhage, cystoid macular edema or macular folds/hypotony maculopathy. The patient can present with pain in case of elevated IOP or angle closure. Anterior rotation of ciliary body results in shallowing of AC more in the periphery. Differential diagnoses include suprachoroidal haemorrhage, mass lesions of the choroid, or RD.

#### Imaging

Ultrasound B–scan helps in delineating the extent of CD and differentiating it from other conditions. Figure 1e corresponds to cilio-choroidal detachment. OCT of the macula can detect subretinal fluid or hypotony maculopathy.

#### Management

Management includes stopping of all topical and systemic IOP lowering agents. Cycloplegics help to deepen the anterior chamber and angle due to posterior rotation of the iris-lens diaphragm. Topical steroids are often required to suppress inflammation [20]. Systemic steroids can be initiated in presence of extensive CD with intense inflammation [20]. Hypotony can be managed either by suturing of trabeculectomy wound, bandage contact lens for a leak, ligation of the tube in case of glaucoma drainage devices. Surgery is indicated in case of a large appositional choroidal effusion (termed “kissing choroids”) as it can lead to vitreoretinal adhesions in the area of apposition and can cause retinal breaks and RD [20]. Other indications for intervention include intractably low IOP with flat anterior chamber, corneal edema, or cataract formation. Inferior-nasal and inferior-temporal quadrant are often selected for drainage of CD. After paracentesis and placement of anterior chamber (AC) maintainer, a conjunctival dissection down to sclera, often in a fornix-based approach, is performed. A rectangular or triangular partial thickness scleral flap is created followed by a 2 mm radial incision 3.5 mm from the limbus carried down to the choroid. Gentle elevation of the sclera and depression of the globe with a cotton swab or muscle hook may be needed to express the choroidal effusion. The scleral incision site is often left unsutured and the conjunctiva is approximated to the limbus [20]. A transconjunctival drainage with the help of trocars has been described in which the trocar should be introduced as flat (ideally 20°) as possible to the scleral plane to avoid choroidal damage [24].

### Haemorrhagic choroidal detachment

Suprachoroidal haemorrhage (SCH) occurs due to rupture of posterior ciliary vessels that bridge the suprachoroidal space from the sclera to choroid, secondary to mechanical trauma due to a rapid reduction in IOP. Incidence is around 0.6% to 1.5% after trabeculectomy and around 0.5–8.3% after tube shunt procedures [25–27]. Risk factors include high preoperative IOP, severe postoperative hypotony, aphakia, pseudophakia, high myopia, anticoagulation, white race, prior intraocular surgery, low postoperative IOP, systemic hypertension, ischemic heart disease, and pulmonary disease.

#### Clinical features

It presents as a smooth, dark brown, dome-shaped choroidal elevation, associated with pain. IOP can be either low or high. Intra operative signs suggestive of SCH include loss of red reflex, hardening of globe and persistent shallowing of AC or even extrusion of intraocular contents when massive.

#### Management

Limited detachments confined to 1 or 2 quadrants, can be observed with conservative management similar to the choroidal detachment [28]. Systemic steroids help in controlling the inflammation caused due to the breakdown of haemoglobin and its by-products [29]. Serial B-scan ultrasonography is important in monitoring the liquefaction and resolution of the SCH (Fig. 1a). Intra-operative SCH requires urgent closure of section and all wounds along with administration of hyperosmotic agents. Isolated massive suprachoroidal haemorrhages can be drained externally using radial sclerotomies, whereas a combined surgical approach including posterior sclerotomies, vitrectomy, sclera buckling and silicone oil injection, may be required in the presence of a concurrent RD [26]. Hemorrhagic choroidal effusion will drain easier if the blood is allowed to liquefy first (which takes place in about 7–10 days), but earlier intervention is performed in intractable elevated IOP or severe eye pain [5]. Technique of drainage is same as that explained for serous CDs.

### Hypotony maculopathy

Pederson described hypotony as an IOP less than 6.5 mmHg, which corresponds to more than three standard deviations below the mean (Statistical hypotony); or where the IOP is low enough to result in a visual loss (clinically significant hypotony) [30]. The reported incidence is 1.3–20% of cases after glaucoma filtering surgery, more commonly after use of Mitomycin-C than 5-fluorouracil [31–33].

#### Etiology

It is the result of reduced scar formation, a direct toxic effect on the ciliary body or certain changes in the conjunctiva. Causes may be wound leak, overfiltration, iridocyclitis, ciliochoroidal detachment, retinal detachment or cyclodialysis. Other risk factors are younger age and myopia. It has also been described following newer supra-choroidal [34] and sub-conjunctival [17] MIGS devices owing to achievement of low post-operative IOP.

#### Clinical features

Patients may be asymptomatic or may have a diminution of vision due to distortion of the overlying retinal receptors and irregular corneal induced astigmatism. Papilloedema, accompanied by retinal vascular engorgement and tortuosity, and wrinkling of the retina and choroid, is the predominant picture (Fig. 1g). Alternate dark and light colored streaks in the fundus are a common finding. Although uncommon, cystoid macular oedema secondary to abnormal capillary permeability has been reported [35]. Gass [36] suggested that Hypotony causes an inward collapse of the scleral wall, resulting in redundancy of the choroid and retina, chorioretinal wrinkling. The retinal folding results due to the peculiar anatomy of the macula. Hypotony causes a decrease in the antero-posterior diameter of the vitreous cavity which causes the very thick perivofeal retina surrounding the very thin foveal retina to be thrown into radial folds around the fovea. Papilloedema occurs due to anterior bowing of the lamina cribrosa, constricting the axonal bundles in the lamina scleralis and reducing axoplasmic transport. Additional source of the disc leak includes leakage from the choriocapillaris, and secondary to hypoxia and endothelial cell damage. Differential diagnoses include idiopathic chorioretinal folds, retrobulbar mass lesions, scleral inflammation, scleral buckle, choroidal tumours, and choroidal neovascularization.

#### Imaging

FFA demonstrates disc leak and fluorescent streaks corresponding to the crest, while the troughs appear as a narrow dark line. B-scan ultrasonography (USG) is useful when the fundus is not easily visualized. It can help in excluding the presence of ciliochoroidal detachment, suprachoroidal haemorrhage and retinal detachment. It shows flattening and thickening of the posterior sclera and choroid. Ultrasonic biomicroscopy can be employed to evaluate the anterior chamber depth, the position of the ciliary body, a cyclodialysis cleft and the presence of anterior ciliary detachment. Intraoperatively, the ciliary body can be directly visualized to evaluate rotation and traction using endoscopy. Optical coherence tomography (OCT) of the posterior pole can help to better demonstrate subtle macular fluid or folds. Magnetic resonance imaging shows an abnormal plaque-like thickening of the macula and flattening of the posterior globe.

#### Management

Management includes identifying the cause and appropriate management. In case of hypotony, make sure all anti-glaucoma medications are stopped. Measures to control wound leak include pressure patching, collagen shield application or contact lens tamponade, fibrin glue application of the wound site, bleb revision or resuturing. In case of over filtering bleb, intra-bleb or subconjunctival peribleb injection of autologous blood, conjunctival compression sutures or bleb revision are options. A modified technique of transconjunctival suturing of the scleral flap has been described by Moster [37]. The suture is taken through the conjunctiva, full-thickness sclera and then out through the conjunctiva. This provides a tamponading effect on the bleb. In late leaking blebs, bleb excision with surgical revision and reconstruction of the filtering bleb with a free conjunctival autograft with or without donor scleral graft can be tried if above mentioned therapies fail. Cataract extraction performed in previously overfiltering eyes can reduce the filtering efficacy by inciting mild inflammation [38].

### Malignant glaucoma

It is alternatively known as ciliary block glaucoma, aqueous misdirection syndrome, and direct lens-block glaucoma. It has been reported to occur in 0.4–6% of cases after penetrating glaucoma surgeries, seen more commonly in chronic angle closure glaucoma [39–41]. Schlenker et al. [17] have reported 4 cases of malignant glaucoma following Xen gel stent. Risk factors include hyperopia, nanophthalmos and female gender (presumably due to smaller anterior segment dimensions) [42].

#### Pathogenesis

It is a multifactorial condition in which there is an alteration in the anatomic relationship of the lens, ciliary body, anterior hyaloid face, and vitreous, resulting in forward movement of the iris-lens diaphragm. Three pathogenic mechanisms have been proposed. (a) Shaffer and Hoskins suggested that due to the posterior diversion of flow, aqueous starts accumulating behind a posterior vitreous detachment resulting in the secondary forward movement of the iris-lens diaphragm [43]. (b) Chandler proposed that a forward movement of lens occurs due to laxity of lens zonules and an increased vitreous pressure [44]. (c) According to Quigley et al., choroidal expansion causes an increased vitreous pressure and the initial compensatory outflow of aqueous along the postero-anterior pressure gradient causes shallowing of the anterior chamber [45, 46].

#### Clinical features

Anterior segment features include shallowing of the central and peripheral anterior chamber associated with increased or normal IOP in the presence of a patent iridotomy (indicating a non-pupillary block mechanism). Posterior segment is usually normal (ultrasound B-Scan should be performed to rule out anterior ciliochoroidal detachment or suprachoroidal haemorrhage which may also present in a similar way). The first symptom is often worsening of distance vision and improvement in near vision secondary to forward shift of the lens iris diaphragm causing a myopic shift. IOP is typically greater than 21 mm Hg, but it may be normal or even low [47]. Pain and inflammation occur when the IOP rises spontaneously and corneal edema develops. UBM may show anterior rotation of the ciliary processes, which press against the lens equator (or the anterior hyaloid in aphakia) and prevent forward flow of aqueous (hence the term ciliary block glaucoma) (Fig. 1d).

#### Management

Management consists of the use of cycloplegics and mydriatics to tighten the zonules thereby pulling the lens-iris diaphragm backwards and deepen the AC; [39] topical antiglaucoma medications and steroid eye drops. Nd:YAG laser capsulohyaloidotomy with disruption of the anterior hyaloid face is often effective in treating malignant glaucoma by establishing a direct communication between the vitreous cavity and anterior chamber [48]. Transscleral cyclodiode laser photocoagulation, argon laser treatment of the ciliary processes or cyclocryotherapy, helps to eliminate an abnormal vitreociliary relationship [49]. Vitrectomy, with or without cataract extraction, along with an additional step of zonulohyaloidectomy, has been described by Tsai et al. [50]. In refractory cases, glaucoma drainage devices are useful to control IOP. Prophylactic measures should be undertaken in high-risk fellow eyes when surgical intervention is planned. Good preoperative IOP control, cessation of miotic drops, prolonged use of cycloplegics after trabeculectomy, avoidance of sudden intraoperative hypotony and anterior chamber shallowing in the postoperative period can help in the prevention of malignant glaucoma [51].

### Vitreous haemorrhage

Law et al. described it in around 5% of patients after aqueous shunt implantation [52]. Recently, it has also been described following Xen gel stent [17]. The haemorrhage may be either due to bleeding from associated posterior segment pathologies like suprachoroidal haemorrhage, retinal breaks, pars plana tube insertion site, deep sclera sutures, or as an extension of anterior chamber bleeds from shunt entrance wound, neovascularisation of iris, iris-tube contact in aphakics. It has also been described after injection of autologous blood into the bleb. Isolated haemorrhages are observed for spontaneous resolution. Vitrectomy is indicated in cases associated with posterior segment pathologies or when vitreous haemorrhage compromises the shunt outflow resulting in increased IOP [53, 54].

### Endophthalmitis

The incidence of bleb-related endophthalmitis (BRE) is around 0.12–1.3%, while bleb infection without vitreous inflammation (so-called blebitis) has been reported in around 0.55–2.6% of eyes [55–57]. Representative cases are shown in Fig. 1b, c. Peri-operative use of anti-fibrotic agents increases the risk (due to thin-walled, avascular, and leaky blebs). Onset is considered to be more insidious than that occurring after cataract surgery, with the initial presentation being a non-specific uveitis [58]. Early infections usually occur within the first month of surgery and are secondary to intraoperative contamination whereas late cases can occur years after glaucoma surgery and are probably due to bacterial contamination through a leaking bleb or tube erosion or exposure. While blebitis responds to intensive topical and systemic antibiotics, its early detection and treatment can prevent the progression to BRE [59]. Treatment of BRE should be aimed at the removal of the infective foci. Thus, it should be managed by a repair of a bleb leak, covering of an eroding tube with a scleral or pericardial patch graft, or removal of the tube/implant. Bacterial strains causing endophthalmitis after shunt procedures are more virulent than those after cataract surgery, with a high percentage of cases being caused by *Streptococcus* species. Thus it has a worse prognosis than the latter and prompt vitrectomy is often considered more beneficial than tap and injections even when presenting vision is better than hand movement [60].

### Retinal detachment

Waterhouse et al. reported an incidence of 5% following Molteno tube implantation, with most cases occurring within 4 months of surgery [61]. An underlying associated pathology can be attributed in most cases, like lattice degeneration, choroidal effusion, retinal apposition from a suprachoroidal haemorrhage, uveitis, chorioretinal scar, trauma, vitreous incarceration, scleral perforation, and retinal dialysis following pars plana tube placement [52]. Management involves treatment of breaks along with the underlying retinal pathology. Careful conjunctival handling is required to prevent bleb leaks, scarring of blebs or exposure/displacements of implants. Silicone oil should be used with caution in the presence of a glaucoma drainage device as it can lead to migration of oil into the subconjunctival space [62]. These patients, in general, have a very guarded visual prognosis.

## Glaucoma after retinal surgery

IOP elevation, either transient or sustained, is a common complication associated with retinal surgeries. It has been reported in around 19 to 28% of cases post surgery [63, 64]. Both external procedure such as scleral buckle [65] and internal procedures such as pars plana vitrectomy [66], intravitreal gas injection [67], intravitreal silicone oil injection [68], intra vitreal steroid injection [69], have been implicated. Anderson et al. [70] reported a 5–12 h IOP spike above 29 mm Hg in 8.4% patients after vitreo-retinal surgery, which included both scleral buckle and pars plana vitrectomy. The cause of raised intra ocular pressure is multifactorial in origin and can occur as a complication of the surgery itself, or due to co-existing primary open angle glaucoma (POAG), angle recession, neo-vascular or steroid induced glaucoma. An association between POAG and retinal detachment has been suggested in view of myopia being a common risk factor [71, 72] and the use of miotics in glaucoma that predisposes to retinal detachment [73]. Certain eye conditions such as Stickler’s, Wagner’s, Retinitis Pigmentosa can also predispose to the development of glaucoma [74].

### Scleral buckle

The procedure of scleral buckle involves the placement of silicone band or sponge against the sclera to relieve the traction over a retinal tear so as to facilitate resolution of sub-retinal fluid. The indentation caused by this on the sclera results in certain changes in the geometry of the eye that predisposes to a secondary angle closure type of glaucoma. The incidence of angle closure glaucoma has been reported to be around 1.4–4.4% [65, 75–77]. It occurs due to anterior displacement of the lens-iris diaphragm due to choroidal congestion. Pavlin et al. demonstrated supraciliary fluid with increased ciliary body thickness by ultrasound biomicroscopy in 12(80%) out of 15 patients after scleral buckle surgery. Obstruction of venous drainage due to the buckle that results in choroidal congestion, culminating in the transudation of fluid into suprachoroidal space, was the proposed mechanism. The result was an anterior rotation of ciliary process with anterior displacement of the lens-iris diaphragm. Thus, it could either lead to a direct angle narrowing or predispose to a pupillary block [78]. Also, an anterior segment ischaemia in the post-operative period, following a high buckle, may be accompanied by an increase in IOP, corneal edema and fibrin in anterior chamber [79]. Reduction in choroidal blood flow can induce visual field changes in open angle glaucoma patients, even without an increase in IOP, similar to that seen in normal tension glaucoma [80].

However, it is important to note that the change in the shape of the globe also leads to an alteration in the distribution of stress and strain, which ultimately decreases the ocular rigidity. Thus, injection of intravitreal gas in these eyes would less likely result in an increase in IOP as compared to an unbuckled eye [81]. This should be kept in mind intra-operatively, so as not to over-fill the globe with gas while attaining a specific IOP.

The aim of treatment should be a control of IOP until the spontaneous resolution of the ciliary body edema occurs, which usually takes several days to weeks. Medical management with topical corticosteroids reduces the inflammation, cycloplegics help to shift the lens iris diaphragm posteriorly and antiglaucoma medications aid in the control of IOP. Miotics should be avoided as it can shift the lens-iris diaphragm forward and increase the inflammation. As the predominant mechanism is a direct angle narrowing rather than a pupillary block, laser iridotomy rarely helps [78]. Laser iridoplasty is usually beneficial in angle closure without a pupillary block [82]. Excess height of the buckle may lead to repeated suprachoroidal fluid accumulation. Loosening or cutting the buckle can help in such cases [83]. When there is permanent synechial angle closure with IOP uncontrolled medically, surgical glaucoma management can be considered. Trabeculectomy becomes challenging in a post buckle scarred conjunctiva, thereby making glaucoma drainage devices a better alternative. Various modifications in placing the implants have been tried. Sidoti et al. reported 85% success rate with the placement of silicone tube in between the buckle and the overlying capsule after incising the capsule over the buckle at the apex [84]. Krupin Denver implant and trimmed Baerveldt implants have been tried with similar end results [85]. Glaucoma drainage devices with an intact plate can also be used, as it has been shown to help in better IOP reduction by increasing the surface area of the fibrous capsule [86].

### Pars plana vitrectomy

**Fig. 2:**
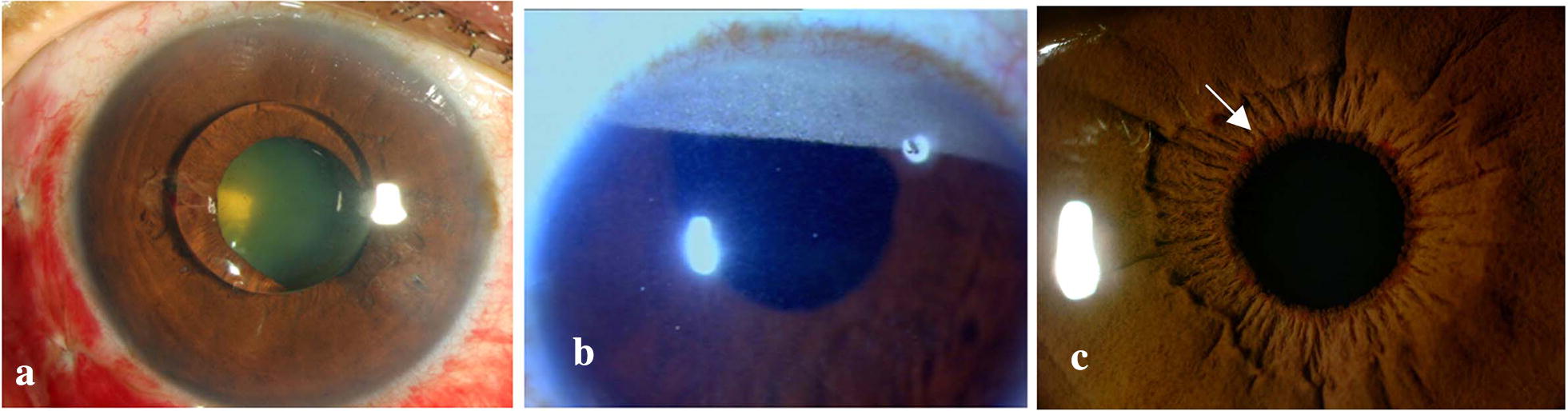
Glaucoma after retinal surgeries: slit lamp photographs showing silicone oil in anterior chamber (**a**), inverse hypopyon (**b**), neovascularisation of iris (arrow) in a patient with neovascular glaucoma (**c**)

The incidence of glaucoma after pars plana vitrectomy has been reported between 11.6 and 20% [66, 87]. Acute post operative increase in IOP can be due to replacement with intra ocular tamponade (Gas/Silicone oil), use of intra operative endo-photocoagulation or post operative fibrinous inflammatory reaction. Late onset glaucoma can be due to topical steroid usage or pre existing causes such as angle recession or neovascular glaucoma (Fig. 2c). Lens extraction has been described as a major risk factor for the development of glaucoma after uncomplicated pars plana vitrectomy [87]. Other risk factors include pseudophakia and family history of open angle glaucoma [88].

Surgical inflammation and debris can reduce the aqueous outflow, alter the biochemical environment due to the release of diffusible factors after removal of cortical vitreous and can increase the susceptibility of optic disc to glaucomatous damage. Chang et al. proposed that in non vitrectomised eyes, there exists a gradient in the oxygen tension in vitreous, the highest being near the retinal surface, decreasing gradually as we reach the anterior vitreous and posterior to the lens. In vitrectomised eyes, oxygen tension near the lens rises to 2–3 times the pre-operative levels. In phakic eyes, this increase in oxygen behind the lens causes nuclear sclerosis, while in pseudophakics and aphakics, this increase in oxygen tension leads to change in the extracellular matrix in trabecular meshwork and thereby reducing aqueous outflow. This explains the increased risk of glaucoma in pseudophakic and aphakic eyes after vitrectomy [66]. Acute rise in IOP has also been demonstrated during minimally invasive vitreo-retinal surgeries [89, 90]. The rise mostly occurs during trocar insertion and can be prevented by modifying the technique of insertion from a direct insertion to a twisting manoeuvre.

Medical management is the first line of therapy for elevated intraocular pressure. When IOP is uncontrolled medically, surgical management can be considered. Due to conjunctival scarring, trabeculectomy is challenging in these cases. Inoue et al. reported the success rate of trabeculectomy with adjunctive Mitomycin-C to be 55.1% at 1 year, decreasing to 43.1% at 3 years. They proposed that high preoperative IOP and neovascular glaucoma are risk factors for failure [91]. Glaucoma drainage implant can be considered when there is a high risk of failure with trabeculectomy or in previously failed trabeculectomy. Rososinski et al. reported similar success rates with pars plana and anterior chamber Baerveldt tube insertions, with no increased risk of complications [92].

### Intravitreal gas injection

Expansile gases like SF6 (sulphur hexa fluoride), C3F8 (perfluoro propane) used in pneumatic retinopexy or combined with pars plana vitrectomy can cause elevated IOP. SF6 doubles its size in 36 h and C3F8 quadruples in 3 days and the greatest pressure elevation occurs at the time of maximum expansion. Incidence of high IOP following intravitreal injection of 20% SF6 and 14% C3F8 has been described to be 6.1% and 18%, respectively [93, 94]. Injection of intravitreal gas causes anterior displacement of the lens-iris diaphragm and secondary angle closure glaucoma and therefore face down position is advised in these cases to prevent the gas bubble from touching the lens and causing anterior pressure. Changes in the atmospheric pressure can cause further expansion of these gases and there by an acute elevation in IOP [95]. These patients are thus advised against a flight travel during the presence of intravitreal gas in the early postoperative period.

Aqueous suppressants can be used to control the intraocular pressure. When increase in IOP causes a compromise in ocular perfusion, aspiration of a portion of intravitreal gas may be needed. When there is pupillary block, laser iridotomy may be required.

### Silicone oil injection

Repeating units of Siloxane (Si–O) constitutes the silicone oil and is one of the commonest substitutes used as internal tamponade in vitreo-retinal surgery.

#### Pathogenesis

Dispersion is the process of splitting of oil into smaller bubbles. These small bubbles have high surface tension and hence tend to coalesce to form a larger bubble. Emulsification is a failure of these bubbles to coalesce to form a larger bubble. Various factors affect the emulsification of silicone oil. It can occur anywhere between 5 to 24 months (mean of 13.2 months) after silicone oil injection [96]. High molecular weight and high viscosity (5000 cSt) oil is more resistant to deformation, dispersion and emulsification. Intrinsic surfactants like fibrinogen, serum, fibrin, blood low density lipoproteins, as well as extrinsic surfactants on surgical instruments and sterilisation agents, decrease the surface tension and increase the risk of emulsification. Presence of nystagmus promotes emulsification while complete fill of globe with silicone oil and presence of a scleral buckle decrease the chance of emulsification [97].

#### Incidence and etiology

Incidence of glaucoma after silicone oil injection ranges from 4.8% and 48% [68, 98, 99]. Early post operative increase in IOP can be due to pupillary block, inflammation, migration of silicone oil into AC (Fig. 2a, b). Late causes include infiltration of trabecular meshwork with silicone oil bubbles, chronic inflammation, synechial angle closure, neovascular glaucoma, migration of emulsified silicone oil into anterior chamber and can also be idiopathic [97]. However, glaucoma can develop without the presence of silicone oil in AC [100] and conversely, normal IOP has been documented in patients with gonioscopic evidence of oil globule in angle [68, 101].

#### Prevention

In aphakic and pseudophakic eyes, it is preferable to perform Ando’s iridectomy inferiorly during surgery to prevent pupillary block. Silicone oil bubble does not enter anterior chamber as it assumes a smooth spherical form due to cohesive forces. When there is pupillary block, aqueous accumulates behind the iris inferiorly in the posterior segment and forces silicone oil into the anterior chamber. An inferior iridectomy prevents such pupillary block. Iridotomy should be at least 150–200 microns to prevent pupillary block [102]. Despite all precautions, it can close in around 32% of cases [103]. Nd YAG laser can be performed in such cases.

Honavar et al. reported pre existing glaucoma, diabetes mellitus, aphakia, rubeosis iridis, silicone oil in the anterior chamber and high IOP on first post operative day as positive risk factors whereas anatomic failure and myopia as negative risk factors for the development of glaucoma. Silicone oil emulsification and Diabetes Mellitus were also associated with poor prognosis for the control of glaucoma [68].

#### Management

Control of IOP with topical and systemic anti-glaucoma medications is usually the first option. Ando’s iridotomy during surgery can prevent pupillary block. When there is pupillary block despite patent iridotomy, it could be due to improper size (too small/too large > 2 mm), location(mid peripheral instead of extreme peripheral) of the iridotomy, or due to recurrence of retinal detachment or development of choroidal detachment [104]. Surgery is indicated when medical management fails. While a simple removal of silicone oil appears to be justified, the timing and its combination with anti-glaucoma surgery remain controversial. Studies have shown that patients treated with silicone oil removal alone may still require anti-glaucoma surgery in the future, whereas a combined surgery may increase the risk of post-operative hypotony [105]. With increasing duration of contact of silicone oil with trabecular meshwork, organic changes can occur in the trabecular meshwork making silicone oil removal alone ineffective to control elevated IOP. Also, elevated IOP can occur even after silicone oil removal due to the splitting of silicone oil droplets into smaller bubbles thus obstructing the trabecular meshwork. Thus, the management needs to be individualised and these eyes need careful and lifelong follow-up.

Conventional filtering surgeries have shown low success rates in these patients. Honavar et al. reported successful IOP control in 3 out of 5 eyes that underwent trabeculectomy with mitomycin C [68]. Emulsified silicone oil can block the ostium in superior trabeculectomy and can cause failure. Glaucoma drainage devices and trans-scleral cyclophotocoagulation can be considered in eyes with extensive conjunctival scarring. Nguyen et al. reported 60% success rate with Molteno implant and failure in 1 eye [106]. Inferior tube placement is preferred to prevent silicone oil blocking the tube [107].

### Intravitreal steroid injection

Intravitreal injection of steroid has found its application in the treatment of macular edema following various conditions like diabetic retinopathy, uveitis, retinal vascular occlusions, choroidal neovascularisation and post cataract surgery. Rise in intraocular pressure is one of the most common complications following intravitreal steroid injection. Incidence has been reported to be as high as 11–79% [108, 109]. The glaucoma is usually of an open angle type. Pre-existing glaucoma, myopia, diabetes mellitus and a family history of glaucoma are important risk factors for the development of glaucoma [110]. The IOP elevation is dependent on the dose, the chemical structure of the steroid and the duration of exposure. Kiddee et al. demonstrated IOP rise in 32% cases after 4 mg intravitreal triamcinolone, 66% and 79% following 0.59 and 2.1 mg fluocinolone implant, respectively, and 11% and 15% after 0.35 and 0.7 mg dexamethasone implant, respectively [108].

#### Pathogenesis

The exact mechanism by which it increases the IOP is highly controversial. Immediate rise of IOP following injection has been attributed to the direct effect of an increase in intra-ocular volume [111]. However, several mechanisms have been theorised for glaucoma developing within 1 week to 2 months. These include an increase in accumulation of glycosaminoglycans in the trabecular meshwork; steroid induced cytoskeletal changes; increased expression of the Trabecular Meshwork Inducible Glucocorticoid Response (TIGR) protein and high levels of Tissue Inhibitor of Matrix Metalloproteinase (TIMP) [110]. The rise in IOP is also high in case of accidental anterior vitreal injection since the drug reaches the anterior chamber in high concentrations resulting in elevated IOP and direct deposition of steroid particles in the trabecular meshwork [112]. Although the upregulation of myocilin gene has been suggested, a study done by Fingeret et al. did not find any co-relation [113].

#### Management

These patients usually respond to topical and systemic anti-glaucoma medications. It is important to monitor the IOP of these patients in the early post operative period so that further injections can be avoided in uncontrolled cases. The IOP decreases as the intravitreal concentration of the drug decreases over 4–6 months. The use of argon laser trabeculoplasty (ALT) [114] and selective laser trabeculoplasty(SLT) [115] have shown promising results. Vitrectomy and removal of an intravitreal implant may be needed in susceptible patients. Filtering surgeries are recommended in cases not responding to laser and medical management.

## Conclusion

Surgical management of glaucoma and retinal pathologies have significantly improved the outcomes of refractory ocular conditions. However, these surgeries are associated with complications and can be a nightmare for the ophthalmic surgeon. Although majority of the above mentioned conditions have a good visual prognosis and are self-limiting, co-existence of 2 or more complications can make the management more complex. Early detection and prompt treatment can help prevent serious sight threatening complications.

## Abbreviations

IOP: intraocular pressure
MIGS: minimally invasive glaucoma surgeries
ODR: ocular decompression retinopathy
AC: anterior chamber
FFA: fundus fluorescein angiography
OCT: optical coherence tomography
CD: choroidal detachment
RD: retinal detachments
UBM: ultrasound biomicroscopy
SCH: suprachoroidal haemorrhage
BRE: bleb-related endophthalmitis
POAG: primary open angle glaucoma
SF6: sulphur hexa fluoride
C3F8: perfluoro propane
TIGR: trabecular meshwork inducible glucocorticoid response
TIMP: tissue inhibitor of matrix metalloproteinase
ALT: argon laser trabeculoplasty
SLT: selective laser trabeculoplasty
